# Polyelectrolyte functionalization of ceria nanozymes: linking dispersion stability and enzyme-like activity

**DOI:** 10.1039/d6na00493h

**Published:** 2026-08-07

**Authors:** Tamás Péter, Dániel Viczián, Viktória Hornok, Nizar B. Alsharif, Şeyma Miray Simav, Meng Mao, Petra Pallagi, Andrea Drubi, József Maléth, István Szilágyi

**Affiliations:** a MTA-SZTE Momentum Biocolloids Research Group, Department of Physical Chemistry and Materials Science, Interdisciplinary Centre of Excellence, University of Szeged 6720 Szeged Hungary szistvan@chem.u-szeged.hu; b Department of Chemistry, Lund University 22100 Lund Sweden; c School of Medicine and Health, Harbin Institute of Technology 150001 Harbin China; d MTA-SZTE Momentum Epithelial Cell Signaling and Secretion Research Group, Department of Translational Medicine, University of Szeged 6720 Szeged Hungary

## Abstract

Cerium oxide (CeNPs) nanozymes represent a versatile class of biocatalytic nanomaterials capable of mimicking multiple enzyme functions, however, their practical application is often hindered by limited colloidal stability. Herein, the interaction of oppositely charged polyelectrolytes, namely heparin (HEP), chondroitin sulfate (CS), poly(acrylic acid) (PAA) and methylglycol chitosan (MGC), with CeNPs was investigated with respect to dispersion stability, peroxidase-like activity and biocompatibility. Distinct charging and aggregation regimes were quantitatively identified over a wide range of polyelectrolyte-to-particle mass ratios. At high surface coverage, adsorption of the macromolecules markedly enhanced resistance against salt-induced aggregation owing to electrosteric repulsion. Surface functionalization also improved the peroxidase-like operation of the CeNPs, with polyanion-coated particles exhibiting substantially higher enzymatic activity than the bare nanozymes. Cell viability assays confirmed excellent biocompatibility of both pristine and functionalized CeNPs, with no detectable cell death upon incubation with the nanozymes. These findings demonstrate that polyelectrolyte adsorption provides an effective strategy to simultaneously improve the colloidal stability and biocatalytic performance of CeNPs, widening their implementation in biomedical and industrial applications.

## Abstract 2

Polyelectrolyte adsorption simultaneously enhances the colloidal stability and peroxidase-like activity of cerium oxide nanozymes, while preserving biocompatibility.

## Graphical abstract

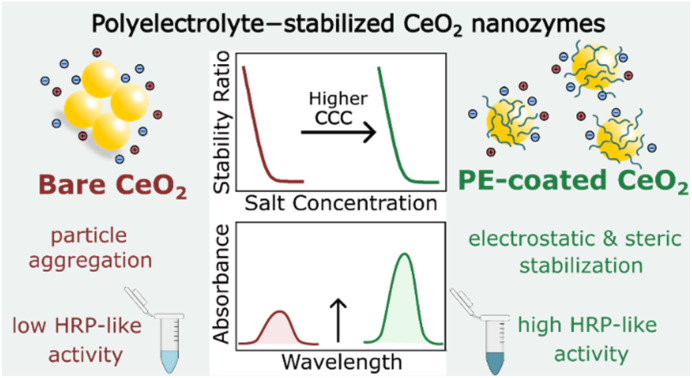

## 1 Introduction

Nanozymes, a class of nanomaterials with enzyme-like catalytic operation, have emerged as promising alternatives to natural enzymes due to their high stability, low cost and tuneable physicochemical properties.^1–4^ Unlike native enzymes, which often lose activity outside narrow optimal temperature or pH ranges, nanozymes can retain enzyme-mimetic functions under harsh conditions.^5,6^ Due to these advantages, extensive research activity focuses on their applications in biomedical, environmental and industrial fields.^7–9^ However, the performance depends on parameters such as particle size, shape and composition, as well as on interface structure, which influence colloidal stability and consequently, catalytic efficiency.^10–12^

Among the broad range of nanomaterials possessing enzyme-like activities, cerium oxide (also known as ceria and denoted as CeNP below) is one of the most widely studied nanozymes.^13–16^ This nanomaterial can mimic the function of several enzymes, such as superoxide dismutase, catalase and horseradish peroxidase utilized in various applications.^17–22^ The multi-enzymatic activity originates from the co-existence of Ce^3+^ and Ce^4+^ ions as well as oxygen vacancies, which enable electron transfer processes.^21,23^ However, CeNPs, similarly to many other metal oxide nanoparticles,^3,4,24^ tend to aggregate into larger clusters due to their high surface energy, which may limit their effectiveness. The aggregation can be induced by changes in the interfacial charge and structure, which are sensitive to alterations in ionic strength, pH or adsorption of surface-active species.^6,16,25^

In general, the colloidal stability of nanoparticle dispersions is governed by the balance of attractive and repulsive interparticle forces acting at the solid–liquid interface.^26^ For instance, van der Waals attraction promotes particle aggregation, whereas electrostatic double layer repulsion arising from surface charge can stabilize dispersions over extended periods.^27^ The classical DLVO (Derjaguin, Landau, Verwey and Overbeek) theory states that the interplay of these forces determines whether particle collisions lead to aggregation or remain reversible.^28–30^ However, certain experimental conditions and dispersion compositions can introduce additional (non-DLVO) interactions, including steric, hydration and depletion forces.^31,32^ Accordingly, the above interparticle forces govern the aggregation behaviour of nanozymes and ultimately, catalytic performance, since the enzyme-mimicking activity is strongly dependent on the available surface area and interfacial environment.^33,34^ Consequently, controlling interaction forces represents a key and important strategy for simultaneously regulating the colloidal stability and functional performance of nanozyme systems.

To prevent aggregation, surface functionalization by polyelectrolytes has proven to be a promising way in various particle systems.^35–38^ Adsorbed species can provide steric stabilization and hence, strong interparticle repulsion leading to highly stable dispersions compared to bare nanozymes.^4,6,25^ Besides, overlap between electrical double layers also prevents aggregation and thus, the combined effect is commonly referred to as electrosteric stabilization.^24,39^ Since CeNPs can be either positively or negatively charged depending on the pH of the samples, it is essential to explore both polyanionic and polycationic stabilizers. As an example, previous studies have demonstrated that adsorption of poly(sodium 4-styrenesulfonate) on CeNPs altered the electrokinetic properties and stabilization mechanisms in dispersions through the formation of polyelectrolyte layers.^40,41^ Such reports enable to identify how the oppositely charged macromolecules interact with the CeNP surface and influence the charging and aggregation behaviour to design stable colloids.

In this work, the stabilization ways of CeNPs were comprehensively explored using polyelectrolytes, while the effect of surface modification on the enzyme-like activity was assessed. Moreover, the resistance of the polyelectrolyte coated nanozymes against salt-induced aggregation and their biocompatibility were also studied. The results provide information for constructing efficient CeNP-based nanozyme systems for applications, where high colloidal stability is a key requirement.

## 2 Experimental section

### 2.1 Materials

Hydrogen peroxide (H_2_O_2_, 30% m m^−1^), dimethyl sulfoxide (DMSO, AnalaR NORMAPUR), HCl (37% m m^−1^, AnalaR NORMAPUR), absolute ethanol (AnalaR NORMAPUR), NaOH (≥99.3%, AnalaR NORMAPUR), ammonia (25%, AnalaR NORMAPUR), methanol (AnalaR NORMAPUR) and ethylene glycol (AnalaR NORMAPUR) were purchased from VWR. The Ce(NO_3_)_3_·6H_2_O (99.5%) was bought from Acros Organics. Polyelectrolytes including methylglycol chitosan (MGC), heparin (HEP), poly(acrylic acid) sodium salt (PAA) were acquired from Sigma-Aldrich, while chondroitin sulfate (CS) was obtained from VWR. The 3,3′,5,5′-tetramethylbenzidine (TMB, ≥99%) substrate for the enzyme tests was bought from Sigma-Aldrich. The solutions were prepared using ultrapure water, which was obtained from an Adrona B30 system. The salt solutions and the water were filtered through a 0.1 µm syringe filter (Millex) to avoid dust contamination. The ionic strength was adjusted by adding KCl during sample preparation.

### 2.2 Preparation of CeNPs

The CeNPs were prepared according to a previously published procedure.^42^ Ethylene glycol (95%, 7.8 mL, 0.12 mol) was dissolved in 92 mL of ultrapure water. Subsequently, 5.16 g of Ce(NO_3_)_3_·6H_2_O was added to the solution, followed by a dropwise addition of ammonia until the pH reached 9.6. The reaction mixture was stirred at 50 °C until a yellow dispersion formed, which was centrifuged at 4200 rpm for 30 min to separate the solid material. Then, it was washed with ethanol and water three times to remove impurities.

### 2.3 Preparation of polyelectrolyte-coated nanozymes (PCeNPs)

The preparation and the characterization of the CeNPs with surface polyelectrolyte layers were conducted under either acidic (pH 4) or basic (pH 10) conditions. The macromolecules used for the functionalization of the CeNPs were oppositely charged to the particles at the given pH. At pH 4, where CeNPs exhibit a positive surface charge, negatively charged polyelectrolytes such as HEP, CS and PAA were used. At pH 10, where the CeNPs bear a negative charge, positively charged MGC was used. To prepare the PCeNP (nanozyme particle with saturated layer of macromolecules on their surface) dispersions (100 mg L^−1^ particle concentration), the CeNPs were mixed with calculated amounts of HEP, CS, PAA or MGC solutions and these stocks were diluted for further measurements.

### 2.4 Electron microscopy

The morphology and size of the CeNPs was analysed by transmission electron microscopy (TEM, Jeol JEM-1400Plus type instrument, Japan). The samples were prepared by drying the dispersions on a copper-coated carbon mesh and 120 kV accelerating voltage was used for imaging in bright-field mode.

### 2.5 X-ray diffraction (XRD)

Powder XRD measurements were carried out using a Philips PW 1830 diffractometer equipped with a Cu anode, operated at an applied voltage of 40 kV and 30 mA cathodic current. The XRD patterns of CeNP powder were recorded over a 2*θ* range of 20–80° and at 5° min^−1^ rate.

### 2.6 Electrophoretic light scattering

Electrophoretic measurements were carried out using a Litesizer 500 instrument (Anton Paar), equipped with a 658 nm laser source, at a scattering angle of 175° and 25 °C. The maximum applied voltage was 200 V during the experiments, which was automatically decreased at elevated ionic strengths due to the higher conductivity of the samples. The electrophoretic mobility (*u*) of each sample was measured five times, averaged and zeta potential (*ζ*) values were calculated using the Smoluchowski equation:^43^1
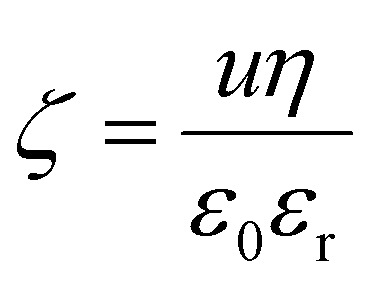
where *η* is the viscosity of the medium, *ε*_0_ is the dielectric permittivity of vacuum and *ε*_r_ is the relative permittivity of water. For sample preparation, calculated volumes of salt and polyelectrolyte solutions were mixed to obtain the desired concentrations and CeNPs were added from a stock suspension to reach a final particle concentration of 10 mg L^−1^ in 2 mL volume. The polyelectrolyte dose was systematically varied between 0.1 and 5000 mg g^−1^ (mg polyelectrolyte per g CeNP). Prior to each measurement, the samples were equilibrated for 2 h.

### 2.7 Dynamic light scattering (DLS)

The rate of particle aggregation was investigated by DLS using an ALV/CGS-3 Compact Goniometer with a 633 nm laser source and at a scattering angle of 90°. The correlation functions were collected for 20 s and 100 runs were performed for each time-resolved experiment. A second order cumulant fit was applied to the correlation function to determine the hydrodynamic radius (*R*_h_). Aggregation behaviour was quantified using the stability ratio (*W*), based on apparent aggregation rate coefficients (*k*_app_), obtained from the time-resolved DLS measurements as:^29^2
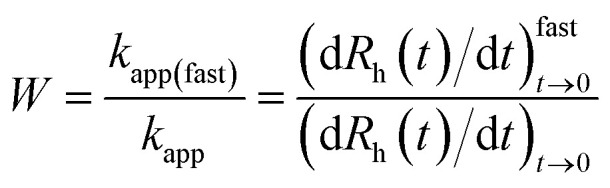
where *t* is the experiment time and the subscript “fast” indicates diffusion-controlled aggregation of the particles achieved at 1 M KCl concentration. Note that the above experimental protocol has an average error of 10%. The destabilizing power of the salts was quantified by determining the critical coagulation concentration (CCC), the electrolyte concentration, at which the system transforms from a stable dispersion (*W* ≫ 1) to a rapidly aggregating (*W* = 1) one, using the following equation:^44^3
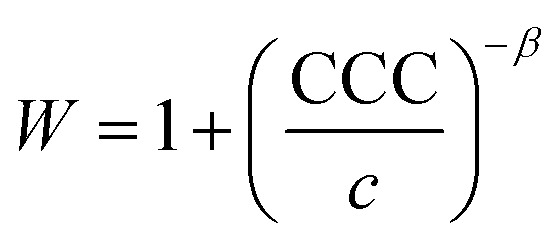
where *c* is the molar concentration of the salt and the value of *β* was derived from the slope of the stability plots in the slow aggregation regime (*i.e.*, before the CCC). For each measurement, 2 mL dispersions were prepared following the same procedure as for the electrophoretic study, except that the DLS experiments were initiated immediately after adding the appropriate volume of the particle stock dispersion.

### 2.8 Horseradish peroxidase (HRP) assay

The HRP-mimicking activity was evaluated based on the oxidation of TMB substrate in the presence of H_2_O_2_, where natural HRP or its nanozyme mimic catalyses the reaction leading to the formation of a blue product with a characteristic absorption peak at 652 nm.^45^ Due to its low solubility in water, TMB was dissolved in DMSO and adjusted to a final concentration of 0.5 mM in each sample. The concentrations of H_2_O_2_, CeNPs and PCeNPs were kept constant. For each reaction, 100 µL of 10 mM TMB was mixed with 200 µL of 500 mg L^−1^ CeNPs (or the corresponding functionalized nanoparticles) and 1640 µL of ultrapure water resulting in a total volume of 1940 µL. Finally, 60 µL of 500 mM H_2_O_2_ was added to initiate the reaction. The UV-Vis spectra were recorded after 30 min reaction time. The assays were carried out at pH 4, where the peroxidase-like activity of ceria nanozymes is known to predominate.^25^ The above experimental procedure leads to an average error of 3% on the reaction rate data.

### 2.9 CellTiter-Glo® 3D cell viability assay

The CellTiter-Glo® 3D cell viability assay is based on the quantification of intracellular adenosine triphosphate (ATP) as an indicator of metabolically active and viable cells. The amount of ATP detected is in line with the number of viable cells present in the culture. The assay relies on a luciferase enzyme catalysed reaction that generates a stable, glow-type luminescent signal. The kit was removed from −20 °C and placed to +4 °C overnight prior to use. The assay buffer was equilibrated at 37 °C for 30 min before the experiment. The HeLa cells were plated onto a white, flat-bottomed microplate at 1 × 10^5^ cell per well density. After 24 h of incubation, the cells were washed with warm sterile HEPES buffer to eliminate the culture media. After the supernatant was removed, the cells were treated with the adjusted concentration of the nanozyme in buffer for 30 min at 37 °C in a humidified cell culture incubator followed by a washing step. Subsequently, 100 µL of HEPES buffer and 100 µL of CellTiter-Glo® 3D reagent were added to the well. The microplates were then transferred to a BMG CLARIOstar Plus multi-mode microplate reader and incubated at room temperature with shaking for 5 min. Luminescence was measured after 25 min activation time. The data were normalized to the initial cell number and relative light unit (RLU) values were then calculated by setting the untreated cell control to 100%.

### 2.10 Statistical analysis

Statistical analyses were performed using GraphPad Prism (version 10.3.1, GraphPad Software). Cell viability data are presented as mean ± standard deviation (SD). Error bars represent the standard deviation of 3 separate measurements/conditions. Differences between groups were analysed using the Kruskal–Wallis test. A *p*-value < 0.05 was considered statistically significant.

## 3 Results and discussions

### 3.1 Bare nanozyme characterisation

**Fig. 1:**
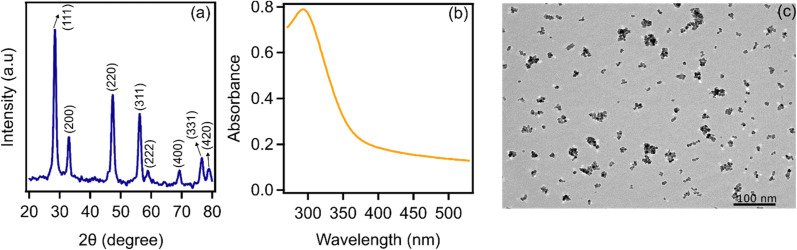
(a) XRD patterns, (b) UV-Vis spectrum and (c) TEM image of the bare CeNPs. XRD and TEM measurements were carried out with dried samples.

The formation of the CeNPs was successfully verified and properties were assessed by various methods. The XRD pattern of the powder sample shown in Fig. 1a exhibits all characteristic diffraction peaks of cerium oxide within the measured 2*θ* range. These reflections are in good agreement with the standard reference pattern (JCPDS card no. 34-0394) of the material and consistent with previously reported results.^25,46^ The UV-Vis spectrum of the CeNP (Fig. 1b) represents a characteristic absorption maximum at 300 nm, in line with earlier studies.^47,48^

Furthermore, the morphology of the CeNPs was investigated by TEM. As shown in Fig. 1c, the sample consists of aggregated nanoparticles with relatively uniform particle sizes. Such aggregates are commonly observed for oxide nanoparticles and mainly attributed to the drying process during sample preparation.^25^

The charge and size of CeNPs were determined in aqueous dispersions. Electrophoretic mobility measurements revealed that they are positively charged at acidic and negatively charged at alkaline pH (see electrophoretic mobility and zeta potential data in Table S1 in the SI), in line with the previously reported point of zero charge of 6.2.^25^ Such a pH-dependent charge is due to the protonation equilibrium of the surface hydroxyl groups.^49,50^ Further experiments were conducted at pH 4 and 10, at which hydrodynamic radii of 124 and 108 nm, while polydispersity indices of 29 and 27% were determined, respectively (Table S1). These data indicate the presence of small clusters of individual particles and a relatively narrow size distribution.

### 3.2 Functionalisation with polyelectrolytes

**Fig. 2:**
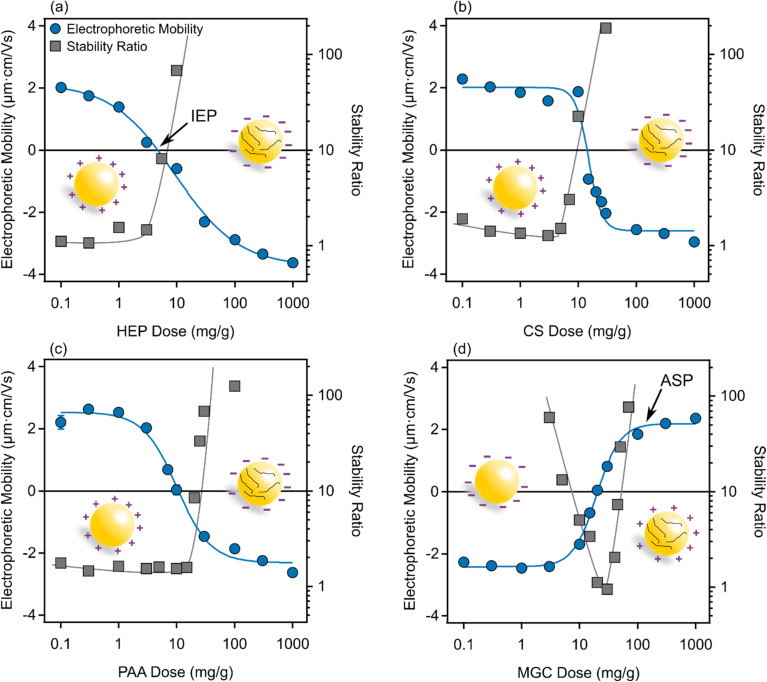
Electrophoretic mobility (circles) and stability ratio (squares) values of the CeNPs at different polyelectrolyte doses at pH 4 (a–c) and pH 10 (d). The ionic strength was kept at 10 mM by addition of KCl to the samples. The concentration of the CeNPs was set at 10 mg L^−1^ and the mg g^−1^ unit refers to mg polyelectrolyte per one gram of CeNP. The lines just serve to guide the eyes.

To determine the colloidal stability regimes (*i.e.*, the conditions, at which slow or fast aggregation occurs) of CeNPs in the presence of polyelectrolytes, particle charge and hydrodynamic size were determined at a wide range of doses. In general, at pH 4, the CeNPs have a positive surface charge and therefore, the addition of oppositely charged HEP, CS and PAA decreased the electrophoretic mobility values due to charge neutralisation (Fig. 2a–c).

At intermediate doses, the isoelectric point (IEP) was reached, *i.e.*, the polyelectrolytes fully neutralized the positive charge of the particles at this concentration. Adjusting higher doses gave rise to charge reversal and formation of negatively charged particles. The electrophoretic mobility values eventually reached a minimum at the onset of the adsorption saturation plateau (ASP) indicating that the CeNP surfaces were fully covered by adsorbed polyelectrolytes. Further addition of polyelectrolytes did not affect the mobility significantly, because the excess remained dissolved in the aqueous medium. The main tendencies in the mobility data were generic, *i.e.*, they were similar in the HEP, CS and PAA samples, while the IEP and ASP values were system specific. These observations are consistent with previous data obtained with poly(sodium 4-styrenesulfonate)-coated ceria nanoparticles, where adsorption-induced charge reversal was also reported.^40,41^

At pH 10, the charging behaviour resembled that observed in the above systems, with the difference that the particles initially had negative surface charge and became positively charged upon the adsorption of MGC (Fig. 2d). The IEP and ASP data for all systems are summarized in Table S2 in the SI.

The aggregation processes were investigated under the same experimental conditions (*e.g.*, particle concentration, pH, ionic strength and polyelectrolyte dose range) as in the electrophoretic measurements. This enabled the establishment of correlation between the charge and aggregation rates of CeNPs. The stability ratios were calculated using eqn (2) from the time dependent change of the hydrodynamic radius (see some examples in Fig. S1). At pH 4, adding polyelectrolytes in low doses (*i.e.*, below the IEP) resulted in stability ratio values close to unity in the presence of HEP (Fig. 2a), CS (Fig. 2b) and PAA (Fig. 2c) indicating that most of the particle collisions resulted in dimer formation. Above the IEP, in parallel with the formation of negatively charged particles due to charge reversal, the stability ratio increased until the ASP, after which highly stable dispersions were observed, since data could not be determined due to the absence of changes in the hydrodynamic radii. These findings clearly support the need of stabilization for CeNPs of positive charge, as they form unstable dispersions without polyelectrolyte functionalization in appropriate doses.

At pH 10, highly negative electrophoretic mobilities are associated with high stability ratios at low doses indicating electrostatic stabilization of the particles under these conditions (Fig. 2d). However, when the MGC concentration approaches the IEP, pronounced destabilization occurs. The stability ratio decreases to one, leading to fast particle aggregation in the dispersions. Further increasing the MGC dose, above the IEP, the aggregation slows down and the MGCCeNPs become stable around the ASP. Similar concentration-dependent destabilization-restabilization behaviour has been previously reported for other oppositely charged colloidal particle-polyelectrolyte samples.^24,28,51^

In all of the above systems, the doses needed for the complete CeNP coating procedures were determined from the mobility data as the intersections of the steep regions near the IEP and the near-constant regions around the ASP, yielding data of 100 mg g^−1^ for HEP, 65 mg g^−1^ for MGC and 60 mg g^−1^ for both CS and PAA (Table S2). Time-resolved DLS measurements confirmed that stable dispersions are formed at these doses.

### 3.3 Salt-induced aggregation and origin of interparticle forces

**Fig. 3:**
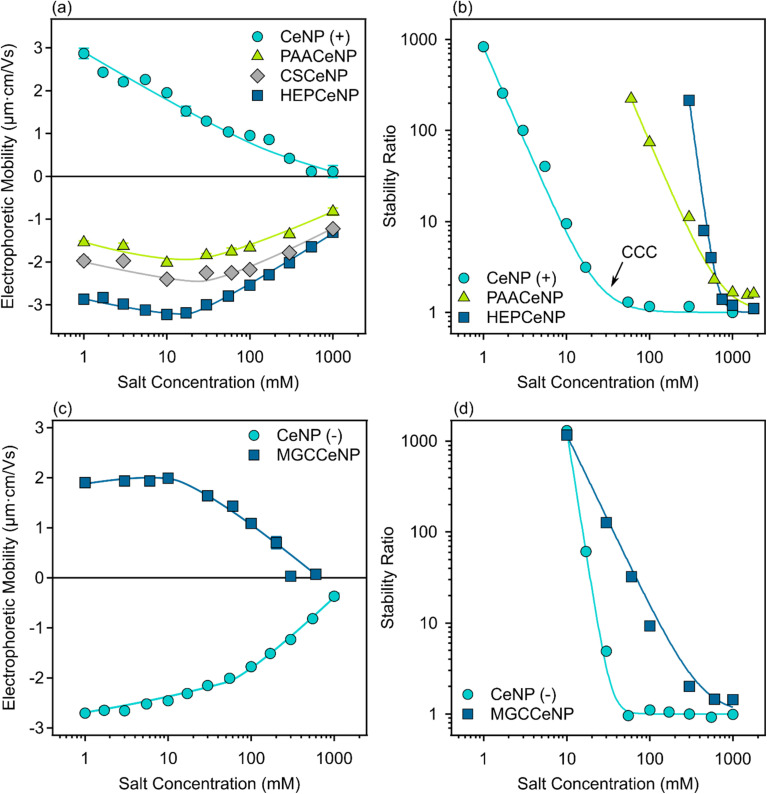
Electrophoretic mobilities (a and c) and stability ratios (b and d) of PCeNPs as a function of the ionic strength adjusted by KCl. The top row corresponds to the pH 4 (positively charged CeNP), while the bottom row to the pH 10 (negatively charged CeNP) experimental conditions. The solid lines for mobility data just serve to guide the eyes, while those for stability ratios were calculated with eqn (3) to obtain the CCC values.

Since salinity exposure is a critical factor in most of the applications, the resistance of all four PCeNPs to salt-induced aggregation was investigated by measuring electrophoretic mobility and stability ratio across a wide range of ionic strengths. At pH 4, increasing ionic strength led to a decrease in the absolute values of the electrophoretic mobilities in all systems due to charge screening by the dissolved salt constituents (Fig. 3a). The bare particles carried a positive charge under this condition, which decreased steeply with increasing ionic strength. The functionalized systems showed similar trends, but their mobility values at the same ionic strength were different. Among them, the particles coated with HEP exhibited the highest negative mobility (Table S2), whereas in the presence of PAA and CS, the magnitude of the mobility values was lower across the entire concentration range. At pH 10, similar trends were observed. Accordingly, bare CeNPs carried a negative surface charge and the electrophoretic mobility values decreased with increasing ionic strength due to electrostatic screening (Fig. 3c). However, lower absolute mobility values were measured in the case of MGCCeNPs, along with a steeper decrease in mobility with increasing salt concentration.

Fig. 3b shows the stability ratios at different KCl concentrations at pH 4. For bare CeNPs, high values were determined at low salt levels indicating stable dispersions. The data decreased with increasing salt concentration and eventually reached unity, corresponding to the fast aggregation phenomenon. These two regimes are separated by the CCC, which was determined by fitting the stability ratio data with eqn (3). Note that the obtained CCC values (listed in Tables S1 and S2) describe the destabilization power of the salt applied.^27^ The aggregation occurs well below the salt concentration, at which the particle charge approaches zero. This observation has been also reported in a previous study.^52^ In the case of functionalized particles, significantly higher CCC values were obtained indicating a strong stabilizing effect of the adsorbed polyelectrolyte layers. The most stable particles were the PAACeNP indicated by the highest CCC value compared to CSCeNP and HEPCeNP. A very similar scenario took place at pH 10, *i.e.*, the MGC coating gave rise to a significant increase in the CCC.

Comparing the CeNP and PCeNP systems, such a difference in the CCC data and in the slopes of the stability ratio curves indicate the presence of non-DLVO repulsive forces, most likely of steric origin.^37,39^ This effect becomes more pronounced in saline environments, where the adsorbed chains adopt a more extended conformation on the particle surface resulting in thicker polyelectrolyte layers and enhanced steric repulsion.^53^ The aggregation rates of PCeNPs in the diffusion-controlled regime (*k*_app(fast)_ in Table S2) were somewhat lower than in the case of the bare CeNPs (Table S1), in particular for MGCCeNP and PAACeNP, which also indicate the effect of the adsorbed polyelectrolyte chains on the speed of particle aggregation.

The slow and fast aggregation regimes separated by the CCCs in the salt-dependent stability ratio data can be qualitatively described by the DLVO theory, while the significant difference between the CCC of CeNPs and PCeNPs is a clear sign of the presence of non-DLVO interactions. To further investigate the nature of the interparticle forces, the charge density of the particles at the slip plane (*σ*, listed in Tables S1 and S2 for CeNPs and PCeNPs, respectively) was calculated from the ionic strength dependence of the zeta potentials using the Debye–Hückel approximation:^54^

**Fig. 4:**
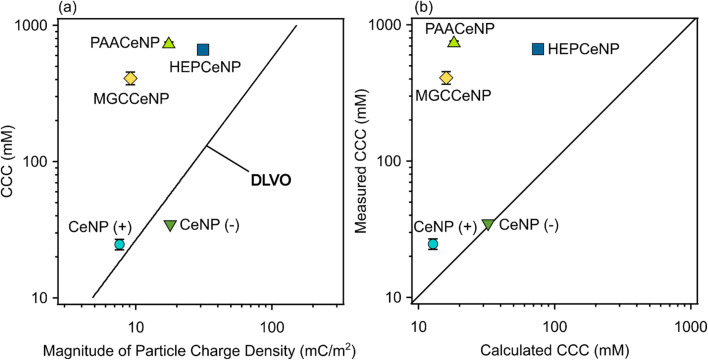
(a) Measured (by DLS, symbols) and estimated (with eqn (5), solid line) CCC data *versus* the particle charge density obtained with eqn (4). (b) Measured (by DLS) and calculated (from electrophoretic mobilities^30^) CCC values and the diagonal line indicates the possible perfect agreement between measured and calculated CCCs. Note that CCC could not be determined experimentally in the CSCeNP system due to the absence of the diffusion-controlled aggregation regime in the ionic strength range studied.

4 *σ* = *ε*_0_*ε*_r_*κζ*

where *κ* is the inverse Debye length. In addition, the CCCs were estimated from the *σ* data with the DLVO theory, assuming that the energy barrier does not completely vanish at the CCC:^33^5
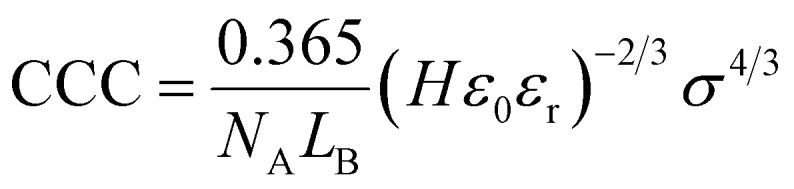
where *N*_A_ is the Avogadro number, *L*_B_ is the Bjerrum length and *H* is the Hamaker constant (a literature value of 2.5 × 10^−20^ J was used^55^). The estimated and measured CCCs at different particle charges are shown in Fig. 4a.

The CCC of the bare CeNPs were close to the DLVO prediction indicating that particle aggregation was mainly driven by electrostatic and dispersion forces in these cases. Besides, the data obtained for the PCeNP systems, such as MGCCeNP, HEPCeNP and PAACeNP, showed the largest deviation from the theoretical curve pointing to significant contribution of non-DLVO steric forces to the stabilization mechanism, *i.e.*, both double layer and steric repulsions contribute largely to the remarkable resistance of these particles against salt-induced aggregation.

The previous findings were further confirmed by obtaining the CCC values from the electrophoretic mobilities based on a recently developed method,^30^ which assumes the presence of solely DLVO forces. Comparing these data to the ones measured by DLS (Fig. 4b), a reasonable agreement was found for the CeNPs indicating again double layer repulsion and van der Waals attraction as major interparticle forces. Nevertheless, the CCCs of PCeNPs did not correlate pointing to the presence of interactions arising from the adsorbed polyelectrolyte chains giving rise to steric repulsion between PCeNPs.

Note that CCC could not be measured for the CSCeNPs by DLS owing to the absence of the fast aggregation regimes until the highest ionic strength applied (1000 mM). However, the mobility (DLVO)-based calculation of the CCC yields 39 mM in this system (Table S2). This again clearly points to the large stabilization effect of the CS adsorbed on the CeNP surface.

### 3.4 Peroxidase-like activity assessment

**Fig. 5:**
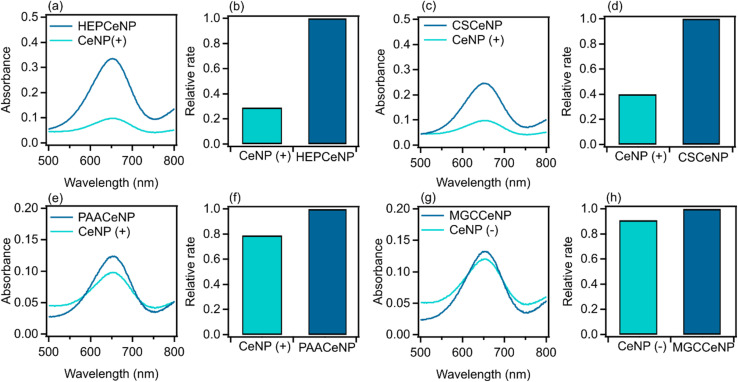
Peroxidase-like activity of CeNPs and HEPCeNP (a and b), CSCeNP (c and d), PAACeNP (e and f) and MGCCeNP (g and h) expressed by the Vis spectra and the relative reaction rates calculated by dividing the absorbance values recorded at 652 nm for the corresponding CeNPs and PCeNPs.

To compare the enzyme-like activities of CeNPs and PCeNPs, the HRP assay^45^ was performed using TMB as substrate (see details in Chapter 2.8). In the presence of the enzyme or enzyme-mimicking substance such as nanozymes, TMB is oxidized by H_2_O_2_ yielding a blue-coloured product with an absorption peak at 652 nm.^56^ The measured absorbance data and the calculated relative reaction rates are shown in Fig. 5.

For the positively charged CeNP, the coated particles exhibited higher peroxidase-like function indicated by larger absorbance values compared to the bare nanozymes. The most pronounced improvement was observed for the HEPCeNP (Fig. 5a and b, followed by CSCeNP (Fig. 5c and d) and PAACeNP (Fig. 5e and f)), which showed about a four-fold higher absorbance compared to the uncoated particles. The negatively charged CeNP displayed relatively weak catalytic activity (Fig. 5g and h). Coating the particles with MGC polycations only leads to a minor increase in TMB oxidation ability indicating that polycation coating has a much weaker effect on enzyme-like activity than polyanion coatings.

These data trends can be associated with a stronger tendency of the positively charged CeNPs to aggregate in the absence of polyelectrolyte functionalization, while the considerable colloidal stability achieved for negatively charged PCeNPs led to elevated enzyme-mimicking ability. A similar enhancement has been reported previously for PAACeNPs, where increased catalase-like activity was also attributed to a higher affinity of H_2_O_2_ to the surface of the nanoparticles resulting in remarkable antioxidant activity.^57^ For CSCeNP and HEPCeNP, the significantly higher TMB product absorbance (compared to PAACeNP) may be a result of the advantageous interactions (*e.g.*, hydrogen bond formation) between the substrate molecules and polyelectrolyte chains. In addition, the improved colloidal stability of the coated particles most likely gave rise to an increase in the accessible catalytic surface area. However, direct experimental evidence for this mechanism could not be obtained from the present experimental results.

It should be noted that CeNPs exhibit multiple enzyme-mimicking activities, including peroxidase-, catalase- and superoxide dismutase-like functions, which relative contributions depend strongly on the reaction conditions, particularly the solution pH. In the present study, the biocatalytic assays were intentionally performed in acidic samples to compare the peroxidase-like activities of bare and polyelectrolyte-functionalized CeNPs under identical conditions. Investigation of catalase- and superoxide dismutase-mimicking abilities was beyond the scope of this work.

### 3.5 Cell viability tests

**Fig. 6:**
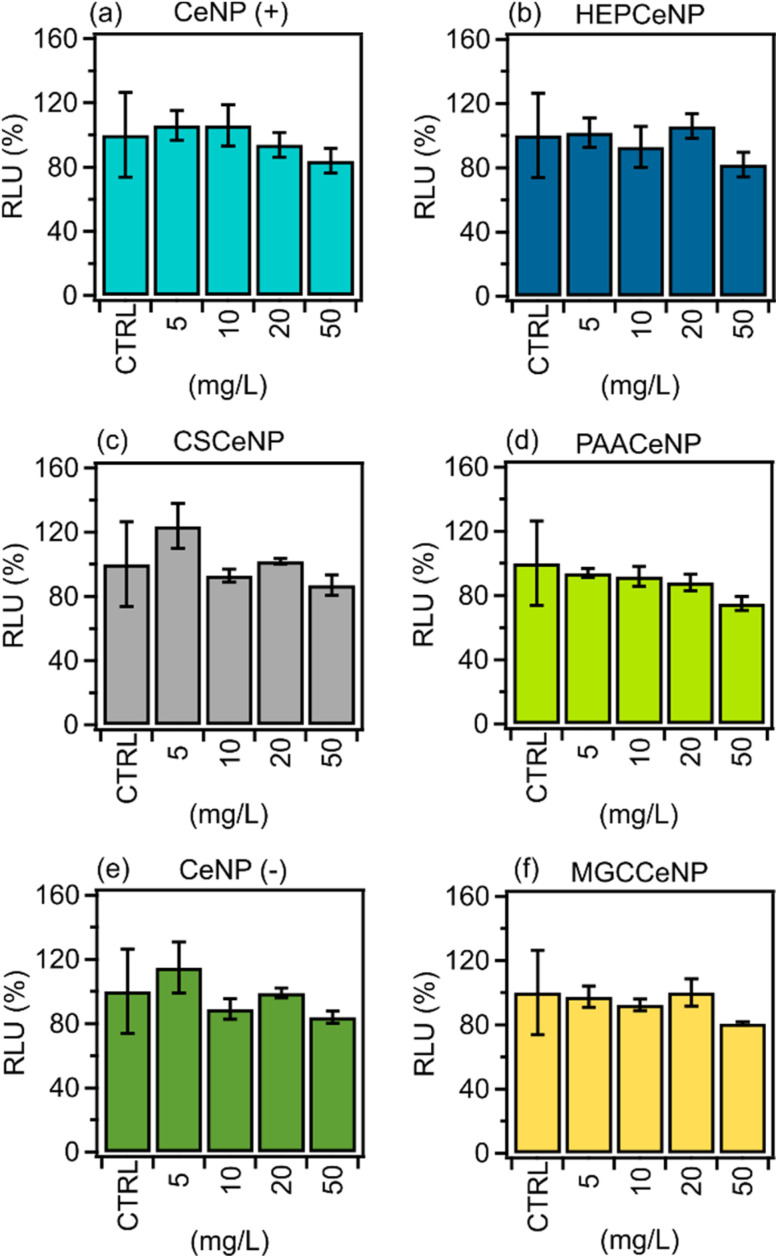
CellTiter-Glo assay for bare positively charged (a) and negatively charged (e) CeNPs as well as for PCeNPs, such as HEPCeNP (b), CSCeNP (c), PAACeNP (d) and MGCCeNP (f). Data were normalized to the initial cell number and RLU values were calculated by setting the untreated cell control to 100%. Bars represent the mean ± SD and *n* = 3/conditions (3 separate measurements/conditions).

To evaluate the biocompatibility of the bare and polyelectrolyte-functionalized CeNPs, CellTiter-Glo luminescent cell viability assay was performed. Briefly, the assay is based on the detection of ATP produced by metabolically active cells. Healthy cells serve as an ATP source for the luciferase-catalysed reaction resulting in a high luminescent signal. The obtained data are presented in Fig. 6.

It was found that none of the investigated systems caused a significant reduction in cell viability compared to the untreated control indicating good biocompatibility of the bare and surface-functionalized nanozymes. The measurements with the PCeNPs gave similar results to the CeNPs confirming that surface functionalization did not cause any additional cytotoxic effects and that this nanozyme family can be applied in applications, in which they are in contact with biological systems.

## 4 Conclusions

This study demonstrates that polyelectrolyte adsorption provides an effective strategy for simultaneously tuning the colloidal stability and enzyme-like activity of CeNP nanozymes without sacrificing biocompatibility. The adsorption of the oppositely charged macromolecules significantly altered the surface charge and aggregation behaviour of the particles resulting in a markedly enhanced resistance against salt-induced destabilization. Analysis of the obtained light scattering data revealed that the stability of the PCeNPs is determined not only by electrostatic double layer interactions described by the DLVO theory, but also by additional steric repulsive forces originating from the adsorbed polyelectrolyte layers. The experimentally determined CCC values were compared with those calculated with the DLVO theory to evaluate the contribution of electrostatic and non-DLVO interactions to the colloidal stability of the dispersions. Remarkable stabilization was observed for all PCeNPs, where electrostatic and steric force contributions jointly resulted in highly stable dispersions. Polyelectrolyte functionalization also enhanced the peroxidase-like activity of the nanozymes, particularly in the case of polyanion-coated particles indicating a strong correlation between dispersion stability, interfacial properties and catalytic performance. Cell viability assays confirmed excellent biocompatibility of both bare and functionalized nanozymes under the conditions investigated. Overall, these findings highlight the importance of controlling interparticle forces and interfacial structure in nanozyme dispersions and establish polyelectrolyte functionalization as a versatile approach for developing stable and highly active CeNP systems for biomedical, environmental and industrial applications.

## Conflicts of interest

The authors declare no conflict of interest.

## Supplementary Material

NA-OLF-D6NA00493H-s001

## Data Availability

The data that support the findings of this study are available from the supplementary information (SI) and from the corresponding author on request. Supplementary information is available. See DOI: https://doi.org/10.1039/d6na00493h.
