# Supplementary material for: Polyelectrolyte functionalization of ceria nanozymes: linking dispersion stability and enzyme-like activity
Source: Nanoscale Adv. 2026 Aug 7;8(18):5267–76. doi: 10.1039/d6na00493h (PMC13479342; doi:10.1039/d6na00493h)
Supplement: Supplementary file 1 [file NA-008-D6NA00493H-s001.pdf]

Supplementary Information (SI) for Nanoscale Advances.
This journal is © The Royal Society of Chemistry 2026

Supporting Information (SI)

Polyelectrolyte Functionalization of Ceria Nanozymes: Linking Dispersion Stability and Enzyme-Like Activity

Tamás Péter,[a] Dániel Viczián,[a] Viktória Hornok,[a] Nizar B. Alsharif,[ab] Şeyma Miray Simav,[a] Meng Mao,[ac] Petra Pallagi,[d] Andrea Drubi,[d] József Maléth[d] and István Szilágyi*[a]

[a]*MTA-SZTE Momentum Biocolloids Research Group, Department of Physical Chemistry and Materials Science, Interdisciplinary Centre of Excellence, University of Szeged, 6720 Szeged, Hungary*

[b]*Department of Chemistry, Lund University, 22100 Lund, Sweden*

[c]*School of Medicine and Health, Harbin Institute of Technology, 150001 Harbin, China*

[d]*MTA-SZTE Momentum Epithelial Cell Signaling and Secretion Research Group, Interdisciplinary Centre of Excellence, University of Szeged, 6720 Szeged, Hungary*

**Corresponding author. Email: szistvan@chem.u-szeged.hu*

Table S1. Size, polydispersity, charge and aggregation data of the CeNPs.

particle	R_h (nm)[a]	PDI (%)[a]	EM (μm·cm/Vs)[b]	ζ (mV)[c]	σ (mC/m^2)[d]	CCC_{exp} (mM)[e]	CCC_{calc} (mM)[f]	$k_{app(fast)}$ (m/s)[g]
CeNP (+)	124 ± 14	29 ± 2	2.9 ± 0.1	36.8 ± 1.4	7.6 ± 1.8	25 ± 2	12.8	0.147×10^{-9}
CeNP (-)	108 ± 6	27 ± 2	-2.7 ± 0.1	-34.7 ± 0.4	-18.0 ± 1.3	35 ± 1	32.5	0.134×10^{-9}

[a]Hydrodynamic radius (R_h) and polydispersity index (PDI) were determined by DLS in stable dispersion at 1 mM ionic strength. [b]Measured by electrophoresis at 1 mM ionic strength. [c]Zeta potential calculated with Eq 1. [d]Particle charge density determined by fitting the salt dependent zeta potential data with Eq 4. [e]Experimental critical coagulation concentration values obtained from the stability ratio data using Eq 3. [f]CCC calculated from the EM values (Fig. 3) using a DLVO-based method (10.3390/colloids4020020). [g]The apparent fast aggregation rate constant measured for CeNP dispersions in 1.0 M KCl solutions.

Table S2. Size, polydispersity, charge and aggregation data of the PCeNPs.

particle	IEP (mg/g)[a]	ASP (mg/g)[b]	R_h (nm)[c]	PDI (%)[c]	EM (µm·cm/Vs)[d]	ζ (mV)[e]	σ (mC/m^2)[f]	CCC_{exp} (mM)[g]	CCC_{calc} (mM)[h]	$k_{app(fast)}$ (m/s)[i]
HEPCeNP	4.6 ± 1.7	100 ± 47.5	183 ± 17	19 ± 3	-2.9 ± 0.1	-36.6 ± 0.4	-31.1 ± 2.1	664 ± 45	75.4	0.123×10^{-9}
CSCeNP	14.1 ± 1.3	60 ± 14.0	171 ± 12	18 ± 3	-2.0 ± 0.1	-25.3 ± 1.1	-23.2 ± 3.5	N/A	39.0	N/A
PAACeNP	10.7 ± 1.8	60 ± 14.1	195 ± 22	20 ± 4	-1.5 ± 0.1	-19.8 ± 0.5	-17.4 ± 2.3	737 ± 33	18.2	0.089×10^{-9}
MGCCeNP	20.5 ± 1.2	65 ± 14.5	133 ± 6	13 ± 3	1.9 ± 0.1	24.4 ± 0.8	9.1 ± 2.1	412 ± 44	15.9	0.093×10^{-9}

[a]Isoelectric point measured by electrophoresis (expressed in dose such as mg polyelectrolyte/g CeNP). [b]Adsorption saturation plateau data were determined from the intersections of the steep and the near-constant regions in the electrophoretic mobility curves. [c]Hydrodynamic radius (R_h) and polydispersity index (PDI) were measured by DLS in stable dispersion at 1 mM ionic strength. [d]Measured by electrophoretic light scattering at 1 mM ionic strength. [e]Zeta potential calculated with Eq 1. [f]Particle charge density determined by fitting the salt dependent zeta potentials with Eq 4. [g]Experimental critical coagulation concentration values obtained from the stability ratio data using Eq 3. It could not be determined for CSCeNP due to the lack of dispersion destabilization under the conditions studied. [h]CCC calculated from the EM values (Fig. 3) using a DLVO-based method (10.3390/colloids4020020). [i]The apparent fast aggregation rate constant measured for PCeNP dispersions in 1.0 M KCl solutions.

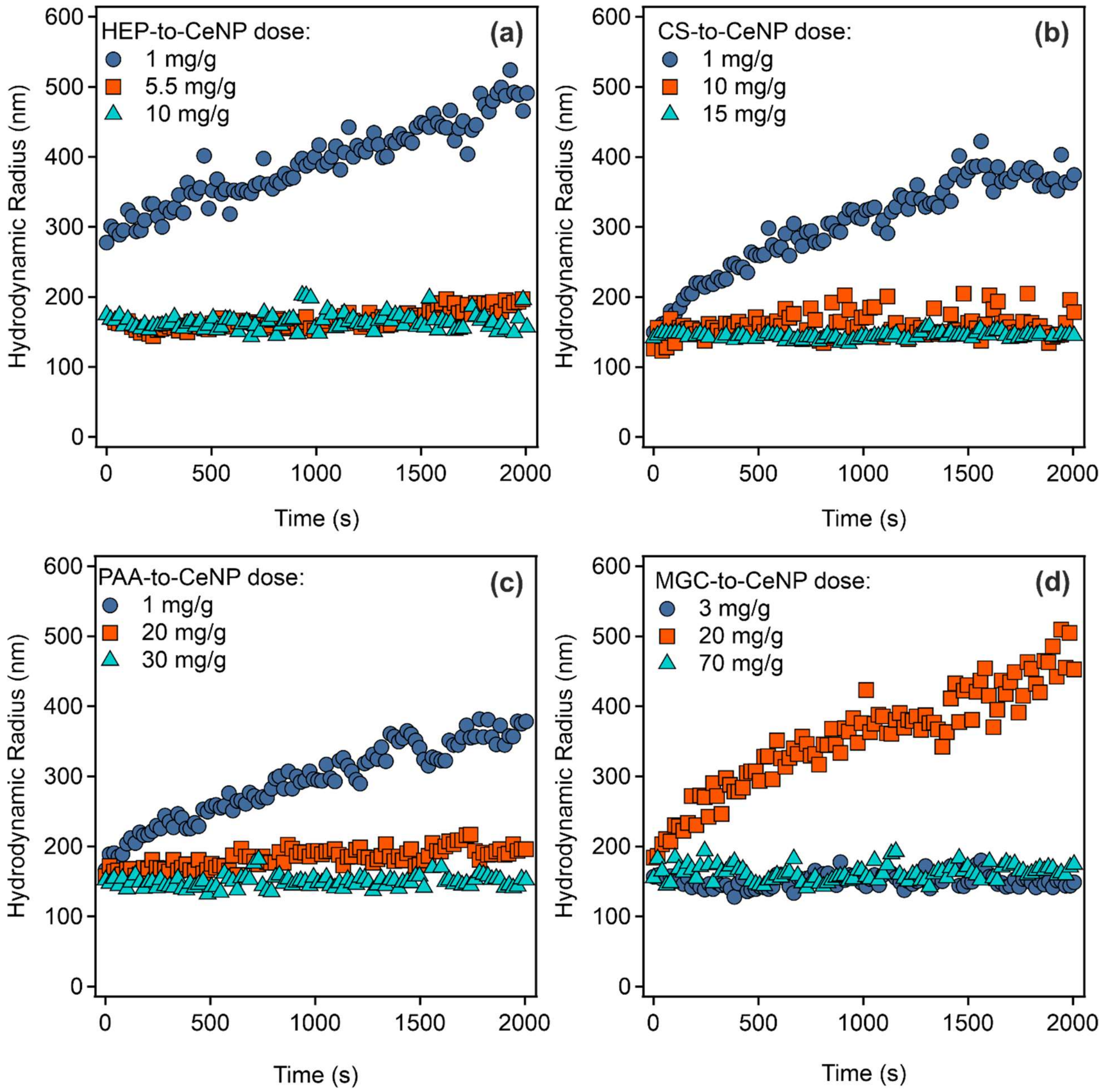

Fig. S1 Time-resolved DLS hydrodynamic size data measured with CeNPs in the presence of HEP (a), CS (b), PAA (c) and MGC (d) at different polyelectrolyte doses. Rapid particle aggregation is indicated by significant increase in the radii, which was experienced near the isoelectric points, while the aggregation slowed down away from this experimental condition.
